# Evaluation of the Performance of the South Africa Regulatory Agency: Recommendations for Improved Patients’ Access to Medicines

**DOI:** 10.1007/s43441-019-00013-5

**Published:** 2019-12-12

**Authors:** Andrea Keyter, Sam Salek, Joey Gouws, Shabir Banoo, Stuart Walker

**Affiliations:** 1 Department of Clinical and Pharmaceutical Sciences, School of Life and Medical Sciences, University of Hertfordshire, Hatfield, UK; 2 South African Health Products Regulatory Authority, Meiring Naudé Road, Brummeria, Pretoria, 001 South Africa; 3 Department of Health, Medicines Control Council, South Africa, Meiring Naudé Road, Brummeria, Pretoria, 001 South Africa; 4 World Health Organization, Appia 20, 1211 Geneva 27, Switzerland; 5 Health Sciences, University of the Witwatersrand, 1 Jan Smuts Avenue, Braamfontein, Johannesburg, 2000 South Africa; 6 Centre for Innovation in Regulatory Science, 160 Blackfriars Road, London, SE1 8EZ UK

**Keywords:** Medicine Control Council (MCC), South Africa Health Products Regulatory Authority (SAHPRA), Review process, Metrics, Milestones

## Abstract

**Background:**

Timely access to new medicines may be addressed through strengthening of registration efficiencies and timelines by establishing and refining value-added registration processes, resources, and systems. The aims of this study were to evaluate the timelines of the milestones of the South African review process and the overall approval process for new active substances (NASs) in 2015–2018 and to provide recommendations for improved patients’ access to new medicines through timely registration.

**Methods:**

Data identifying the milestones and overall approval times for NASs registered by the South African Agency during 2015–2018 were collected and analyzed.

**Results:**

The most NASs (42) were approved in 2017 and the least (15) in 2018. The shortest median approval time (1218 calendar days) was achieved in 2015 and the longest (2124 days), in 2018. All applications were reviewed using the full review process, and 16/99 (16%) were assigned priority status and were reviewed and approved through the fast track review.

**Conclusions:**

While the extensive delays in NASs approvals in South Africa may be attributed to inefficient operational processes, resource constraints, and as an increased number of applications for registration, the newly established South African Heath Products Regulatory Agency has re-engineered and streamlined its regulatory review process, which has been piloted and will be enhanced prior to final implementation. Among recommendations for improvement, SAHPRA should consider measurement and monitoring of milestones, facilitated regulatory pathways, implementing a reliance strategy, and a quality management system.

## Introduction

National regulatory authorities (NRAs) are mandated to ensure the quality, safety, and efficacy of medicinal products [1–3]; however, the World Health Organization (WHO) has reported that one-third of the world’s population does not have timely access to such products [4]. Roth and associates have suggested that the lack of timely access to new medicines may be addressed through the strengthening of registration efficiencies and timelines by establishing and refining value-added registration processes, resources, and systems [5]. Keyter et al. evaluated the South African regulatory review process, as it had been applied by the Medicines Control Council (MCC), prior to the establishment of the South Africa Health Products Regulatory Authority (SAHPRA) [6]. While this study provided an indication of the overall timelines for new active substances (NASs) approved and registered by the MCC during 2015–2017, it focused on the organization and the regulatory review process of the MCC and the status of good review practices that had been implemented by that organization [6].

This study aimed to identify the key milestones of the review process and to evaluate review times in South Africa for NASs approved during 2015–2018. This review is the first to be carried out of the specific milestones and timelines embedded within the South African regulatory review of NASs, as it had been applied by the MCC between 2015–2017 as well as through the transition period of the MCC to SAHPRA during 2018.

### Study Objectives

The main objectives of this study were to

- identify the key milestones and measure the timelines of the South African review process for the period 2015–2018;
- evaluate the overall timelines for the different new medicines approved in South Africa during the period 2015–2018; and
- review the challenges and opportunities for expediting the overall review timelines to enhance the regulatory performance in South Africa with a view to improving patients’ access to new medicines.

## Methods

### Data Collection Process

Data were collected reflecting the timelines between the various milestones, including dossier validation and queue time, scientific assessment as well as the overall approval times for NASs, including new chemical entities (NCEs), biologicals, and major line extensions (MLEs) registered by the South African NRA during the period 2015–2018. The data were sourced directly from the directorate within the Authority responsible for recording the timelines required to complete the regulatory review process. The number of NASs registered during this period was validated against the notifications of registration of medicines published by the Authority in the *Government Gazette* and available in the public domain. The definitions of the application types included in the study are shown in Table 1.

**Table 1.** Definitions of the Application Types Included in the Study.

Application Type	Definition
New active substances (NASs)	Applications including new chemical entities, biologicals, and major line extensions
New chemical entity (NCEs)	Applications for medicinal products that have not previously been approved by the MCC or SAHPRA; this includes chemical and radiopharmaceutical substances that have not been previously available in South Africa for the cure, alleviation, treatment, prevention, or in vivo diagnosis of diseases in humans and animals
Biological medicines (Biologicals)	Applications for medicinal products where the active ingredient and/or key excipients have been derived from living organisms or tissues, or manufactured using a biological process. Biological medicines can be defined largely by reference to their method of manufacture (the biological process) and include applications that require additional scientific assessment by the Biological Medicines Committee of the MCC or SAHPRA [7]
Major line extension (MLEs)	Applications for medicinal products, already registered by the MCC or SAHPRA, where a change to the registered medicinal product, is sufficiently great that it cannot be considered to be a simple variation to the original product, but requires a new product authorization. Such changes include major new therapeutic indications or new disease states, extension to new patient populations (e.g., pediatric patients), a new route of administration, or a novel drug delivery system
Fast track	Applications that are eligible to be assigned to a fast track status in order to expedite the registration of essential medicines. While the review process is the same for fast track applications, these applications would be prioritized over existing applications, queued for allocation to reviewers

*MCC* Medicines Control Council, *SAHPRA* South African Health Products Regulatory Authority.

### Data Analysis

Data collected during the period 2015–2018 were analyzed and the characteristics of the medicinal products submitted to the authority for registration were described. The review type (fast track/standard) applied to each application was identified (Table 1) as well as the origin (multinational company/local company) of the submission and the definition of the milestones within the review process (Table 2). The median timelines for each of the milestones within the review process as well as the median overall approval times were calculated and analyzed. Median approval times by product type and therapeutic area were determined and all data were analyzed as calendar days.

**Table 2.** Definition of the Milestones Within the Review Process.

Milestones	Definition
Overall approval time	The time between the date stamped on **receipt of dossier** when received by the authority and the **date that marketing authorization** was granted
Dossier validation and queue time	The time between the date stamped on **receipt of dossier** and the **date of allocation** of the dossier to a reviewer
Scientific assessment time	Amount of time spent actively reviewing the dossier or additional information provided from the **start of scientific assessment** to **completion of scientific assessment**
Applicant time^a^ (clock stop-start time)	Time during which the clock was stopped during the review while the authority awaited responses or additional data from the company
Other regulatory authority time	Time taken up by the authority during the review for administration from the **completion of scientific assessment** to the date of **marketing authorization granted**

^a^Data pertaining to applicant time was not available.

## Results

The characteristics and number of the NASs approved (NCEs, biologicals and MLEs) are shown in Table 3. While the data for the period 2015–2017 represent the performance of the MCC, the results described for 2018 reflect the performance of SAHPRA during the initial stages of its establishment and transition. However, the results for 2018 do not reflect the re-engineered, streamlined processes developed by SAHPRA that are still in the process of being piloted prior to their final implementation. The NRA registered a total number of 121 NASs during 2015–2018. The applications for NASs registered during this time were submitted by 22 multinational companies and 6 local companies. The results of this study will be valuable in providing a baseline to quantitatively reflect the improvements that are envisaged through the implementation of the finalized, enhanced SAHPRA regulatory review process.

**Table 3.** Categories of New Active Substances Approved (2015–2018).

Submissions	Year of Submission				(2015–2018)
	2015	2016	2017	2018	Total
Number approved (NASs)	31	33	42	15	121
Number of approved NASs submitted by multinational companies	23	27	33	10	93
Number of approved NASs submitted by local companies	8	6	9	5	28

Type of NASs Approved*	Year of Submission				(2015–2018) Total
	2015	2016	2017	2018	
NCEs					
Regular review	16 (15;1)	24 (19;5)	31 (25;6)	12 (7;5)	83
Fast track review	8 (2;6)	3 (2;1)	5 (4;1)	0	16
Biologicals					
Regular review	3 (3;0)	6 (6;0)	5 (3;2)	3 (3;0)	17
Fast track review	0	0	0	0	0
MLE					
Regular review	4 (3;1)	0	1 (1;0)	0	5
Fast track review	0	0	0	0	0

*Number of applications submitted (number of applications submitted by multinational companies; number of applications submitted by local companies).

### Milestones and Timelines in the Regulatory Review Process

**Figure 1.**
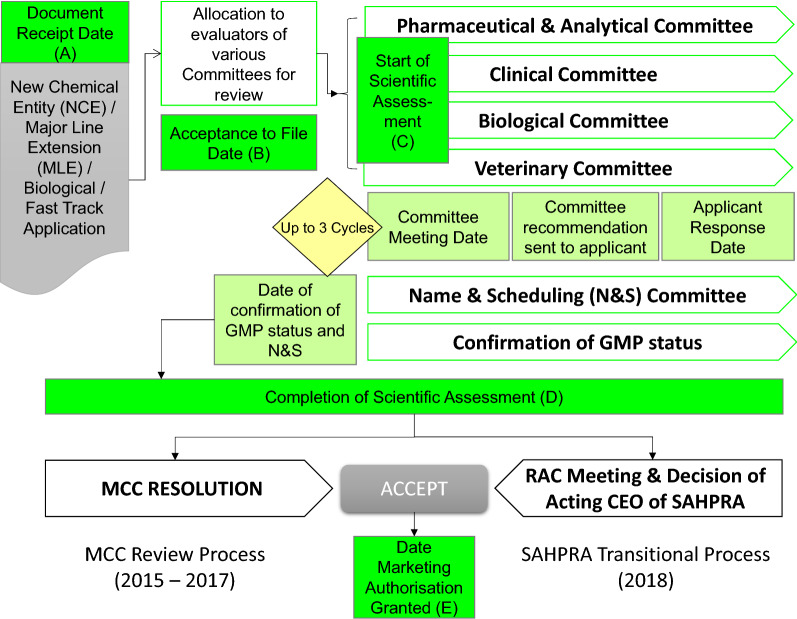
Regulatory Review Process* of the Medicines Control Council and South African Health Products Regulatory Authority’s transitional process. *GMP* Good Manufacturing Practice, *MCC* Medicines Control Council, *RAC* Regulatory Advisory Committee, *SAHPRA* South African Health Products Regulatory Authority.

The milestones in the MCC review process (2015–2017) are similar to those identified by other NRAs and are reflected in Fig. 1a–e.

Applications for registration are received and the dossier receipt date (A) recorded. Each application undergoes administrative and technical screening against the evaluation criteria published in the various guidelines prepared by the authority and made available in the public domain. Following this validation of the application, the acceptance to file date (B) is recorded and the application would be allocated to a reviewer for evaluation. The date of allocation of the application to either an internal or external reviewer is recorded and considered to be the start date of the scientific assessment (C). Following the initial assessment of the application the reviewer prepares an assessment report which is tabled for discussion at the relevant scientific committee meeting. The assessment report is reviewed and discussed at the relevant committee meeting and a recommendation is made. Scientific committee meetings are typically planned in 6–8 week cycles. There is no limit to the number of committee cycles an application could go through. The committee either prepares a recommendation to the company requesting further information to support the registration of the product or a final recommendation supporting the approval or rejection of the product. Companies are required to provide a response to the committee’s request for additional information within 180 calendar days. Once all the relevant scientific committees have made a final recommendation, the date for the completion of the scientific assessment (D) is recorded.

Up until this point, the review process applied previously by the MCC and the transitional review process applied by SAHPRA in 2018 were the same. Under the MCC review process (2015–2017), the final recommendation of the various committees would be tabled for ratification at a Council meeting. A Council resolution would then be prepared and if the resolution supported the registration of the product, a marketing authorization would be granted. The date of the Council meeting at which the Council resolved to register the product was recorded as the date when marketing authorization (E) was granted.

Under the transitional SAHPRA review process (2018), recommendations of the various scientific committees are considered by a regulatory advisory committee (RAC) which advises the Chief Executive Officer (CEO) of the Authority on the approval or rejection of an application, in line with the amended provisions of the Medicines and Related Substances Act, 1965 (Act 101 of 1965) [8]. As such, the SAHPRA CEO now has the responsibility for carrying out the functions of the authority, including regulatory decisions to approve or reject an application for the registration of a medicinal product, as described in Section 3 (4)(e). Section 39 of the Act allows the CEO to appoint relevant committees to advise on all registration and regulatory matters.

### Overall Approval Times

**Figure 2.**
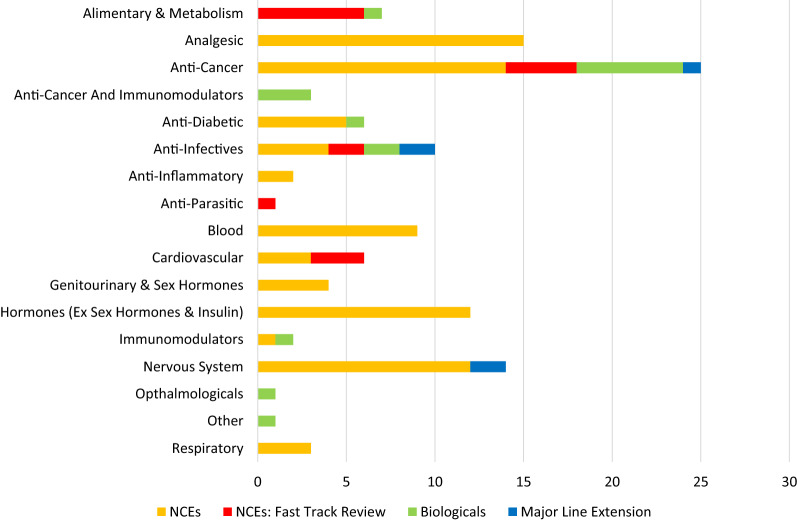
Categories of New Active Substances Approved by Therapeutic Area (2015–2018).

The NASs approved by the MCC (2015–2017) and SAHPRA (2018) covered 16 common therapeutic areas of which oncology products (*n* = 25; 14 NCEs, 4 fast track; 6 biologicals; 1 MLE) were the highest followed by analgesics and anti-infectives (Fig. 2). The results showed that the largest number of NAS approvals (*n* = 42) were recorded in 2017 and that the majority (*n* = 36) approved were NCEs (Table 3). All the NAS applications (*n* = 121) that were registered during 2015–2018 were reviewed by the authority using the full review process. Sixteen NCEs were assigned priority status and were reviewed through the fast track review process, while no applications for biologicals or MLEs were processed through this route.

**Figure 3.**
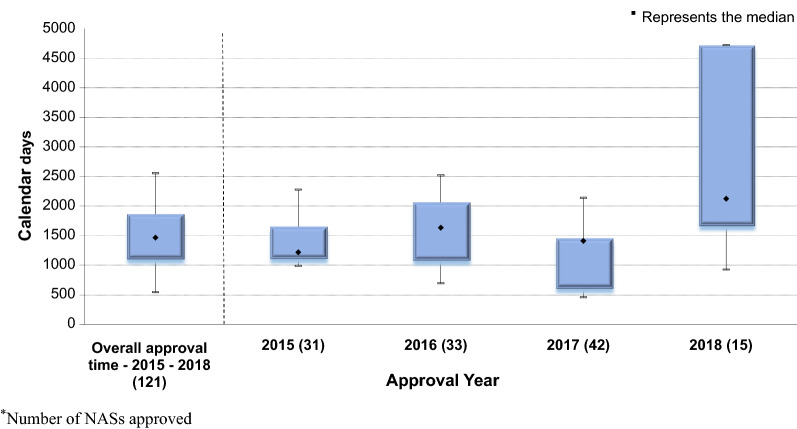
Median Overall Approval Times* for New Active Substances (2015–2018).

The overall median approval time for NASs was 1466 calendar days, and this included NCEs evaluated through the standard and fast track review process as well as biologicals and MLEs approved between 2015 and 2018 (Fig. 3). Furthermore, the shortest median approval time, of 1218 calendar days, was achieved in 2015 and the longest median approval time, of 2124 calendar days, was recorded in 2018. Most NASs (*n* = 42) were approved in 2017 and the least number of NASs (*n* = 15) were approved in 2018.

### Approval Times for New Chemical Entities and Biologicals

**Figure 4.**
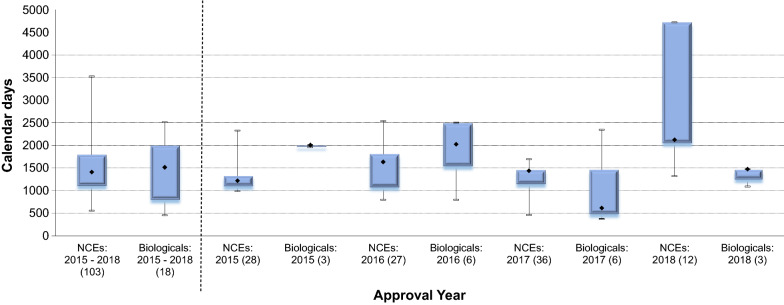
Median Overall Approval Times for New Chemical Entities and Biologicals (2015–2018).

During 2015 and 2016, the median overall approval timelines were less for NCEs (1175 and 1726 calendar days, respectively) when compared with biologicals (2010 and 2027 calendar days, respectively) (Fig. 4). In 2017 and 2018, the median overall approval timelines for biologicals decreased (725 and 1476 days, respectively) and was less than that observed for NCEs (1466 and 2124 days, respectively). The shortest median overall approval time achieved during this period was for 6 biologicals approved in 2017 (725 calendar days). The longest median overall approval time (2124 calendar days) was observed for 12 NCEs approved in 2018.

**Figure 5.**
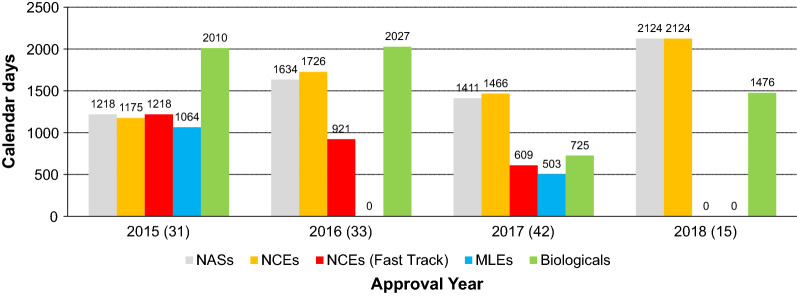
Median Overall Approval Time for New Actives Substances by Categories, (2015–2018).

Three biologicals and 16 NCEs were approved in 2015, 8 NCEs were approved through the fast track review process and the four MLEs approved were also for NCEs (Fig. 5). There were no MLEs approved in 2016 or 2018. Only 1 MLE, which was a biological, was approved in 2017. During the SAHPRA transitional period of 2018, no applications for registration were assigned fast track status. The fast track review process was applied to 3 NCEs approved in 2016 and 5 NCEs approved in 2017. Overall, this study demonstrated that over the period 2015–2018, the review times for NCEs significantly increased, from 1175 days (2015) to 2124 days (2018), while for biologicals this decreased from 2010 days in 2015 to 1476 days in 2018.

## Discussion

National Regulatory Authorities globally measure overall approval timelines for the registration of medicines to demonstrate their performance as regulators. While this metric is not the only indicator of regulatory performance, it does contribute significantly to achieving the mandate of the NRAs in ensuring timely access of safe, quality, and effective medicines to patients. As such, it is critical to any improvement to ensure the routine and accurate measurement and monitoring of performance metrics of the regulatory review process. Benchmarking milestones currently used by NRAs typically include the times for receipt and validation, scientific assessment, applicants’ response, and market authorization to be granted as well as the time taken to complete all administrative activities [9]. The data collected from the MCC and SAHPRA for the period 2015–2018 demonstrated that several of these milestones were recorded, but not measured and monitored.

The authority conducted a full assessment for each of the applications registered during the period 2015–2018. This type of review requires the scientific assessment of the quality, safety, and efficacy data submitted by the company to support the approval of the medicines on the South African market. While the dossier receipt date and date of allocation of the dossier to a reviewer were recorded, it was not possible to confirm the time taken to validate the document through administrative and technical screening. Consequently, it could not be determined how long each application spent in the queue prior to being allocated to a reviewer. While there was no set target for the completion of the scientific assessment, reviewers were requested to complete assessments within 90 calendar days; however, this timeline was not systematically monitored and the data collected demonstrate that this timeline was not always met. Each application was evaluated in parallel by the various scientific committees and the dates of the scientific committee meetings, at which the reviewer’s assessment reports were discussed, were available. There was no limit to the number of times an application could go through a scientific committee cycle. The data collected during the period 2015–2018 reflect that, on average, there was a maximum of three cycles for an application within any given scientific committee. While applicants were encouraged to respond to the request of the scientific committees for additional information within 180 calendar days, this requirement was neither monitored nor enforced. Unfortunately, the data provided did not allow for the accurate calculation of the clock stop so it was not possible to determine the amount of time the applications spent with the scientific committee nor the time it took for the applicant to respond. Based on the data collected and reflecting on the correspondence from companies, the consequent assessment report dates, and the committee meeting dates, it is apparent that the authority routinely accepted responses from companies that considerably exceeded the recommended response timeline of 180 calendar days. Nevertheless, if a mandate to reduce company response time were implemented, this could reduce the time that an application would spend in the system.

The review process of the former MCC as well as that during the transitional period for SAHPRA did not include set targets for milestones within the review process and no target was set for the overall approval time of applications. It is critical for NRAs to develop, maintain, and strengthen a culture of performance measurement so that the results can be used to optimize regulatory outcomes.

### Regulatory Review Approval Timelines

The overall approval timelines for the regulatory review achieved by the MCC (2015–2017) and by SAHPRA (2018) are extensive and do not contribute to ensuring timely access to medicines for patients in South Africa. Keyter et al. previously described both the historical and operational factors that have contributed to these extended timelines [9]. While there are currently no comparative studies available to reflect the regulatory performance of South Africa relative to other African countries, it has been noted that a target overall approval timeline of 330 calendar days has been set by the Zazibona collaborative process [10], a harmonization and joint-review initiative in the Southern African Development Community (SADC) region, in which South Africa has participated since 2016. This target is almost five times less than the median approval timeline for NASs reported in this study. The scope of Zazibona includes NASs and is not limited to the assessment of generic medicines, although this is predominantly the group of products currently being reviewed. It also raises the question as to whether applicants wishing to register medicines in South Africa may prefer to opt for a registration through the Zazibona pathway in order to circumvent the longer review timelines for NASs demonstrated in the present study.

The Centre for Innovation in Regulatory Science (CIRS) studied the median approval times for NASs approved during 2013–2017 in developing markets and demonstrated that the timelines achieved by South Africa were the longest when compared to those in other developing markets [11]. The timelines reported for South Africa were nearly double when compared with Egypt and China (for whom the second and third longest timelines were reported, respectively), and approximately seven times longer when compared with Mexico (for whom the shortest timeline was reported) [12]. It is, however, important to note that while these results demonstrate vast differences in the overall approval time achieved by South Africa in comparison to other developing markets, many of these countries have implemented facilitated regulatory pathways. These pathways allow NRAs to reduce duplication of regulatory effort, recognize the decisions made by other NRAs, and apply abridged review or verification processes in their assessment of applications for registration of NASs. All the applications for NASs registration approved by South Africa during this period underwent a full review, although alternative Facilitative Regulatory Pathways are now being considered by SAHPRA going forward.

Keyter et al. studied the review times for NASs for South Africa in comparison with NRAs in Australia, Canada, Singapore, and Switzerland and found that the South African overall review timelines were substantially longer [13]. The median approval time for NASs achieved from 2008–2017 by the NRAs in Australia, Canada, Europe, Japan, Switzerland, and the United States were approximately four times faster than that achieved by South Africa under the former MCC [11]. These NRAs, except for Australia, also approved applications for NASs through a priority review process and achieved expedited approval approximately four times faster than the South African priority review process. While the regulatory review times achieved by the MCC and SAHPRA during 2015–2018 are not competitive when compared with the timelines achieved through regulatory pathways available in Africa, other developing markets, and NRAs such as the United States Food and Drug Administration (FDA) or the European Medicines Agency (EMA), there is now an opportunity for transformation and significant improvement.

### Challenges and Opportunities for Improvement

Historically, the MCC did not identify key milestones within the review process and did not set or enforce target timelines for these milestones. The median overall approval time for the registration of NASs was neither measured nor monitored and this, together with a growing number of applications, resulted in a large backlog in medicine registration. At its inception, SAHPRA’s inherited backlog of work comprised approximately 16,000 applications, including 8300 registration applications and 7200 variation applications. Over 90% of these applications were for generic medicines and included duplicate applications as well as applications for products with multiple strengths. Of these, approximately 545 were applications for the registration of NASs [14]. An application survey was concluded in January 2019 and an analysis of the information provided through this survey resulted in the agreed withdrawal of approximately 3000 registration applications from the backlog [14]. A validation exercise was completed in consultation with the industry stakeholders to facilitate the planning of the backlog work schedule and to define the process and timelines for resubmission of updated applications for registration [14]. The work plan will be devised to support the prioritization of applications for medicines serving the therapeutic areas that address the highest public health need within South Africa, as agreed upon in consultation with the South African National Department of Health [14]. A dedicated team will be appointed by SAHPRA to address the backlog, in an effort to avoid resource constraints or delays in its routine workload [14]. The backlog clearance program has been planned for implementation in the third quarter of 2019 and it is the intention of SAHPRA to clear the backlog within 2 years [14]. Median overall approval times recorded for 2015–2018 demonstrated a noteworthy departure from the approval times achieved by other NRAs of a similar size and with a similar regulatory mandate. All of the NASs approved during this period were evaluated using a full review. The regulatory effort applied in the assessment of applications for registration should be commensurate with the level of risk of the product and should not impose an unwarranted regulatory burden.

The use of facilitated regulatory pathways is supported through Section 2B(2b) of the Medicines and Related Substance Act, 1965 (Act 101 of 1965) and should be considered in order to ensure the effective allocation of limited resources [8, 15]. Participation in joint and shared review initiatives will continue to support the effort to decrease the overall approval time for medicine registration [6, 16]. While the former MCC had set a target review time of 250 calendar days for products reviewed using the fast track review process, this target was not achieved during the period 2015–2017. The SAHPRA should define the eligibility criteria for fast track designation and should consider the possibility of stratifying the pathways and target timelines within the fast track process to accommodate breakthrough therapies that demonstrate substantial improvement over available medicines or accelerated approvals for NASs addressing unmet needs or in response to emergency situations, as applied by other NRAs such as the United States FDA [17]. This stratified approach may also require SAHPRA to consider regulatory trade-offs involving acceptance of surrogate end-points supported by strengthened post-marketing commitments such as the reallocation of regulatory resources from pre-marketing to post-marketing functions [5, 17]. As SAHPRA moves forward with the implementation of the newly restructured review process, it is critical to ensure that the quality management system is formalized to support the consistent application of good regulatory, review, and reliance practices within the review process [6, 9, 13]. Furthermore, in an effort to prove itself as an effective, responsive, transparent, and accountable regulatory authority, SAHPRA should consider the use of the universal methodology for benefit-risk assessment of NASs and progressive quality decision-making practices [6, 9, 13, 18, 19].

## Conclusions

This study has evaluated the regulatory review process of the former MCC as well as that applied by SAHPRA during the initial stages of its establishment and transition. The key milestones and timelines of the South African review process for the period 2015–2018 were identified and measured and the challenges and opportunities for decreasing the overall approval timelines together with an improved review process have been considered. While the extensive delays in NAS approvals could be attributed to deficient operational processes, resource constraints, and increased volume of applications for registration, there is now an opportunity for improvement. The SAHPRA have developed a re-engineered, streamlined regulatory review process that has been piloted, and will be improved prior to final implementation.

The following key recommendations may be considered to support the restructuring and enhancement of the SAHPRA regulatory review process:

*Measuring and monitoring* Identify, record, monitor, and measure milestones in the review process, and codify and enforce benchmarked targets for each milestone.

*Facilitated regulatory pathways* Define and codify the type of product review assessments that will be used by SAHPRA, including full, abridged, and verification reviews, and continue to enhance regional, continental, and international collaborations for joint and shared reviews.

*Regulatory trade-offs* Consider surrogate end-points to inform expedited market authorization for NASs supported by strengthened post-market surveillance commitments.

*Robust information and communications technology system* The development, implementation, and maintenance of enhanced ICT solutions, supported by dedicated resources, should be considered in order to facilitate the adequate and accurate tracking of applications and decision making as well as improved document management, transparency, and stakeholder communication.

*Quality management system* Formalize good regulatory, review, and reliance practices within the review process, implement a universal methodology for benefit-risk assessment, and ensure transparent and consistent quality decision-making practices.
